# Schools’ Participation in the Community Eligibility Provision Affects Students’ Receipt of Emergency Benefits during the COVID-19 Pandemic

**DOI:** 10.3390/nu14224919

**Published:** 2022-11-21

**Authors:** Francesco Acciai, Punam Ohri-Vachaspati, Michael J. Yedidia

**Affiliations:** 1 College of Health Solutions, Arizona State University, Phoenix, AZ 85004, USA; 2 Center for State Health Policy, Institute for Health, Health Care Policy and Aging Research, Rutgers University, New Brunswick, NJ 08901, USA

**Keywords:** community eligibility provision, Pandemic Electronic Benefits Transfer, free or reduced-price meal eligibility

## Abstract

Pandemic Electronic Benefits Transfer (P-EBT) benefits were intended for families of school children who lost access to free or reduced-price school meals (FRPMs) during the COVID-19 pandemic-related school closures. In high-poverty communities, all students from schools participating in the Community Eligibility Provision (CEP) were automatically eligible for P-EBT benefits; in non-CEP schools, only students already participating in FRPMs—for which their parents submitted an individual application—were eligible for P-EBT benefits. Using publicly available data from 105 K-12 public schools located in 4 sizeable low-income New Jersey (NJ) cities, this study investigated the association between school CEP participation status and the reach of P-EBT benefits for eligible students. A generalized linear model with a logit link, a binomial family, and robust standard errors was used. Across all levels of FRPM eligibility based on students’ household income, as expected, almost all students from CEP schools received P-EBT benefits; significantly fewer received P-EBT benefits if they attended non-CEP schools, even when they were eligible for FRPMs. Our findings show that without changes to the qualification process for CEP, large numbers of eligible children will not receive the intended health benefits of federal meals programs or similar emergency relief initiatives. Expanding CEP eligibility and simplifying the process through which schools qualify would likely improve the uptake of federal meals programs and emergency interventions, and more effectively achieve their intent.

## 1. Introduction

Prior to the COVID-19 pandemic, 30 million school-age children participated in the USDA’s National School Lunch Program (NSLP) and over 14 million participated in the School Breakfast Program (SBP) every day [1]. Students participating in school meals programs have healthier diets, are less food insecure, and are better equipped to learn [2]. To encourage participation in school meals, the USDA introduced the Community Eligibility Provision (CEP) as part of the 2010 Healthy, Hunger-Free Kids Act. CEP allows schools that serve predominantly low-income students to provide school meals to all enrolled students at no cost, without requiring individual applications from parents. Even though participating in CEP reduces the administrative burden associated with collecting applications for free or reduced-price meals (FRPMs) and may increase revenue for school meals programs, a large proportion of eligible schools (30% nationally and 48% in NJ in 2020 and 2021) do not participate in CEP [3].

COVID-19 restrictions compelled immediate federal action to address the challenges of providing meals to students in the face of extended school closures. For instance, the US Congress approved the provision of the Pandemic Electronic Benefits Transfer (P-EBT) program, which allowed state agencies to provide financial assistance for food purchases to households that had lost access to FRPMs for their children because of school closures [4]. All students who received FRPMs were eligible to receive P-EBT benefits. Therefore, all students in CEP schools were eligible for P-EBT benefits, whereas only those students whose parents individually qualified them for FRPMs were eligible for P-EBT benefits in non-CEP schools. This analysis compares the P-EBT distribution immediately after the onset of the pandemic across schools with similar levels of students eligible for FRPMs but with different CEP statuses.

## 2. Materials and Methods

### 2.1. Data Sources

We examined the distribution of P-EBT benefits during the first 6 months of the COVID-19 pandemic using data from all public schools located in 4 large NJ cities (i.e., Camden, New Brunswick, Newark, and Trenton) with predominantly lower-income populations (26–36% of individuals living below poverty in 2021) [5]. De-identified data on the distribution of P-EBT benefits were obtained from the New Jersey Department of Human Services. Data were aggregated to calculate the total number of children who obtained the benefits at a school level.

CEP participation status (SY 2019–2020) for all public schools in the study cities was obtained using Open Public Records Act requests from the New Jersey Department of Education.

The school-level characteristics were obtained from the Common Core of Data for SY 2019–2020 from the National Center for Education Statistics (NCES). The main variables of interest from the NCES data were total school enrollment and total number of students eligible for FRPMs. These two variables were used to calculate the proportion of students eligible for FRPMs. Other variables drawn from the NCES were the race/ethnicity of enrolled students and the school level (elementary, middle, or high school).

### 2.2. Statistical Analysis

The analyses were limited to 105 public schools that had complete information on CEP participation status and P-EBT distribution and were conducted using Stata 17. To model the proportion of students who received P-EBT benefits, a generalized multivariable linear model with a logit link, a binomial family, and robust standard errors [6] was used. The predictors were CEP status (yes/no) and the proportion of students who were eligible for FRPMs. An interaction between the two predictors was added to examine the differential association between the proportion of FRPMs and the proportion of P-EBT benefits by school CEP status. Adding other school-level controls—specifically, the race/ethnicity proportion and a binary indicator for primary vs. secondary (middle or high) schools—did not modify the results. After running the model, the *margins* post-estimation command returned the predicted proportion of students enrolled who received P-EBT benefits by school CEP status at different levels of FRPM eligibility. This study was deemed to be exempt by the Arizona State University and Rutgers Institutional Review Boards as it was entirely based on aggregate-level, de-identified, publicly available data.

## 3. Results

Less than half (44%) of the schools in the sample participated in CEP (Table 1), despite high levels of FRPM eligibility in both CEP (71%) and non-CEP schools (83%). Most schools in the sample, regardless of CEP status, were elementary schools. The most prevalent racial/ethnic groups were Hispanic (especially in CEP schools) and non-Hispanic Black.

**Figure 1:**
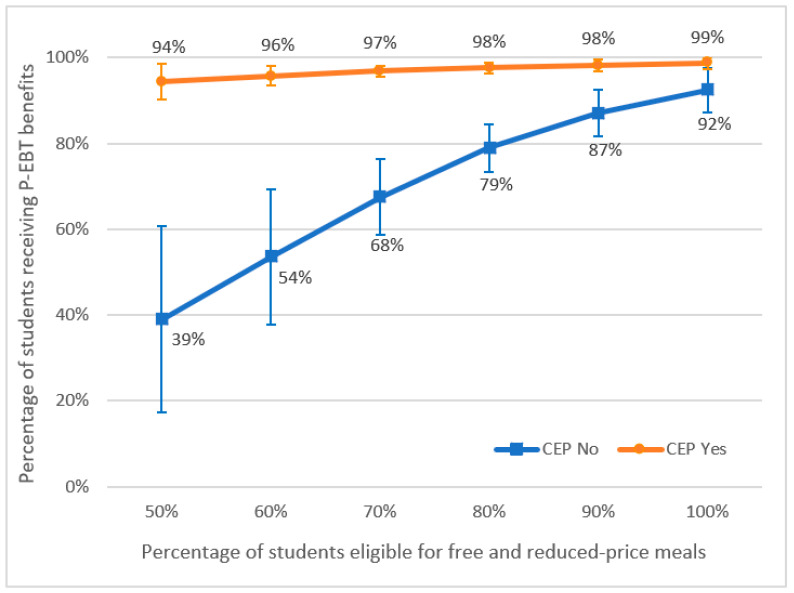
Pandemic Electronic Benefits Transfer (P-EBT) receipt by Community Eligibility Provision (CEP) participation status of the schools.

Figure 1 shows the predicted percentage of students receiving P-EBT benefits based on CEP participation status and the levels of FRPM eligibility of their schools, which was calculated from a generalized multivariable linear model using post-estimation commands. Across all FRPM eligibility levels, almost all students from CEP schools received P-EBT benefits, whereas the proportion of students who received P-EBT benefits in non-CEP schools was significantly lower and consistently below the proportion of students eligible for FRPMs.

## 4. Discussion

At the onset of the pandemic, a lack of access to school meals likely contributed to a rapid increase in food insecurity among families with children [7,8]. The provision of P-EBT benefits—a key USDA response to the pandemic—reduced the food hardship of children by 30% in the week following the disbursement of the benefit [9]. A recent econometric study concluded that the P-EBT program was a cost-effective strategy for addressing food insecurity [10]. Other qualitative research has shown that parents were highly appreciative of P-EBT benefits as they helped offset the cost of meals that their children would have normally received at school [11,12,13]. Our analysis showed that while almost every student attending a CEP school received P-EBT benefits, a significantly lower proportion of students attending non-CEP schools received such benefits. This was not an unexpected result; in fact, it showed that CEP worked precisely as it was intended to. Nonetheless, our findings highlight the magnitude of the inequity resulting from the process of qualifying children for this crucial entitlement, as the likelihood of receiving P-EBT benefits during the pandemic depended on whether a student attended a CEP school rather than a non-CEP school. Notably, this disparity existed at all levels of FRPM eligibility; even when 100% of the students were eligible for FRPMs, there was a higher proportion of P-EBT benefits received by students in CEP schools than in non-CEP schools (99% vs. 92%, respectively). This suggests that expanding CEP eligibility and participation may help increase the reach of emergency assistance programs to families with the highest needs, contributing to reducing disparities in food access and improving health equity [14]. A significant number of students attending non-CEP schools missed out on the P-EBT benefits despite being eligible for FRPMs. Differential P-EBT reach by school CEP status underscores the importance of new federal-level strategies for increasing CEP participation if the intended benefits of P-EBT, or similar policies in response to an emergency, are to be realized. For instance, there are marked state-level differences in the rate of CEP participation among schools eligible for CEP, with rates ranging from 14% in Nebraska to 93% in Vermont [15]. Given the benefits to students associated with CEP participation of schools, ensuring that all eligible schools participate in CEP will constitute an important step toward improving the wellbeing of students.

The recently introduced Child Nutrition Reauthorization bill—the Healthy Meals, Healthy Kids act—includes a provision for summer EBT cards to ensure families have access to resources aimed at providing nutritious meals for children when schools are closed during the summer. However, the process of qualifying for benefits replicates the experience of P-EBT distribution; as a result, unless efforts are made to increase CEP participation among eligible schools, a significant number of children from low-income families will miss out on benefits that can improve their nutrition and wellbeing simply because their CEP-eligible school did not participate in CEP.

One limitation of the current study was that it was based on a sample of New Jersey public schools located in predominantly lower-income areas; therefore, the findings may differ in other contexts or states. Additionally, while we had data on the receipt of P-EBT benefits, we did not have information on how families actually utilized these benefits.

## 5. Conclusions

Expanding CEP eligibility and simplifying the process through which schools qualify would likely improve the uptake of federal meals programs and emergency interventions that rely on school meals eligibility criteria.

## Figures and Tables

**Table 1:** Descriptive characteristics of the schools included in the analysis by CEP status (*n* = 105).

	CEP		*p*-Value ^1^
	No (*n* = 59)	Yes (*n* = 46)	
School Level (%)			0.744
Elementary	74.6	71.7	
Middle/High	25.4	28.3	
School Race (%)			0.000
Hispanic	45.8	65.7	0.023
Non-Hispanic Black	47.2	32.1	
Non-Hispanic White/Other	7.0	2.2	
Total Enrollment	688	611	0.342
School Free or Reduced-Price Meals (%)	82.9	70.7	0.000

^1^ *p*-Values were derived from *t*-tests for continuous variables and from chi-squared tests for categorical variables.

## Data Availability

The data used in the current paper were publicly available and obtained from three different sources: (1) the New Jersey Department of Human Services (through a direct request to staff members); (2) the New Jersey Department of Education (through an OPRA request); and (3) Common Core of Data from the National Center for Education Statistics (from their website: https://nces.ed.gov/ccd/). Accessed on 12 September 2022.
